# Mazabraud Syndrome: A Case Report With 23 Years of Follow-Up

**DOI:** 10.7759/cureus.85972

**Published:** 2025-06-13

**Authors:** Fotios A Tilkidis, Dimitrios I Gelalis, Ioannis K Koumoulidis, Daphne J Theodorou, Stavroula J Theodorou, Ioannis S Gkiatas, Dimitrios V Kosmas, Emilios E Pakos, Ioannis D Gelalis

**Affiliations:** 1 Department of Orthopaedic Surgery and Traumatology, University Hospital of Ioannina, Ioannina, GRC; 2 Radiology, Magnetic Resonance Imaging-Computed Tomography (MRI-CT) Unit, University Hospital of Ioannina, Ioannina, GRC; 3 Radiology, Musculoskeletal Magnetic Resonance Imaging (MRI) Unit, University Hospital of Ioannina, Ioannina, GRC

**Keywords:** complex medical history, differential diagnosis, fibrous dysplasia (fd), intramuscular myxoma, mazabraud syndrome

## Abstract

Mazabraud syndrome is a rare condition characterized by the coexistence of fibrous dysplasia (FD) and intramuscular myxomas. A 46-year-old woman, initially diagnosed with polyostotic FD at the age of 23, developed a palpable mass on her left arm 15 years later, which proved to be an intramuscular myxoma. A diagnosis of Mazabraud syndrome was made. Over the following two decades, she developed additional intramuscular myxomas. The patient denied surgical excision of the soft tissue lesions and was managed conservatively for symptomatic FD. Radiologic imaging, including MRI and ultrasound, played a crucial role in the diagnosis and the assessment of disease progression. Although both FD and myxomas are benign musculoskeletal abnormalities, close follow-up of the patients is essential to monitor changes in the number, distribution, and extent of lesions that may occasionally undergo malignant transformation. This case underscores the importance of early detection and long-term follow-up of patients with FD who may present with, or eventually develop, intramuscular myxomas, comprising Mazabraud syndrome.

## Introduction

Mazabraud syndrome is a rare non-hereditary primary bone dysplasia characterized by the association of monostotic or polyostotic fibrous dysplasia (FD) with single or multiple intramuscular myxomas. This benign disorder was reported by Henschen in 1926, and it was discussed by Girard and Mazabraud in 1957 and later as fibrous dysplasia involving bones and tendons [1-3]. In the literature, Mazabraud syndrome has an incidence of one case per one million people [4], and almost 70% of the reported cases have occurred in women. Because Mazabraud syndrome is rare and the lesions in bone and soft tissue are heterogeneous, diagnosis is challenging and not infrequently missed.

Skeletal FD is uncommon and can be either monostotic or polyostotic. It is characterized by the replacement of bone marrow with fibro-osseous tissue, which undergoes variable degrees of ossification. In 75% of the cases, FD is monostotic and typically affects the long bones, the ribs, and the skull [5]. Interestingly, intramuscular myxoma, a benign myxoid soft tissue tumor without malignant potential, has a reported incidence identical to Mazabraud syndrome, with a 57% female predilection [6-8]. Clinically, myxoma occurs most often as a solitary, painless, ovoid, and slowly growing lesion in the musculature of the thighs [5,9]. When multiple myxomas occur, however, they are universally associated with monostotic or polyostotic FD, known as Mazabraud syndrome. In this report, we describe the clinical presentation and the imaging appearances guiding the management of a female patient with polyostotic FD, in whom the subsequent discovery of painful, enlarging soft tissue intramuscular myxomas over two decades of follow-up established the diagnosis of Mazabraud syndrome.

## Case presentation

**Figure 1:**
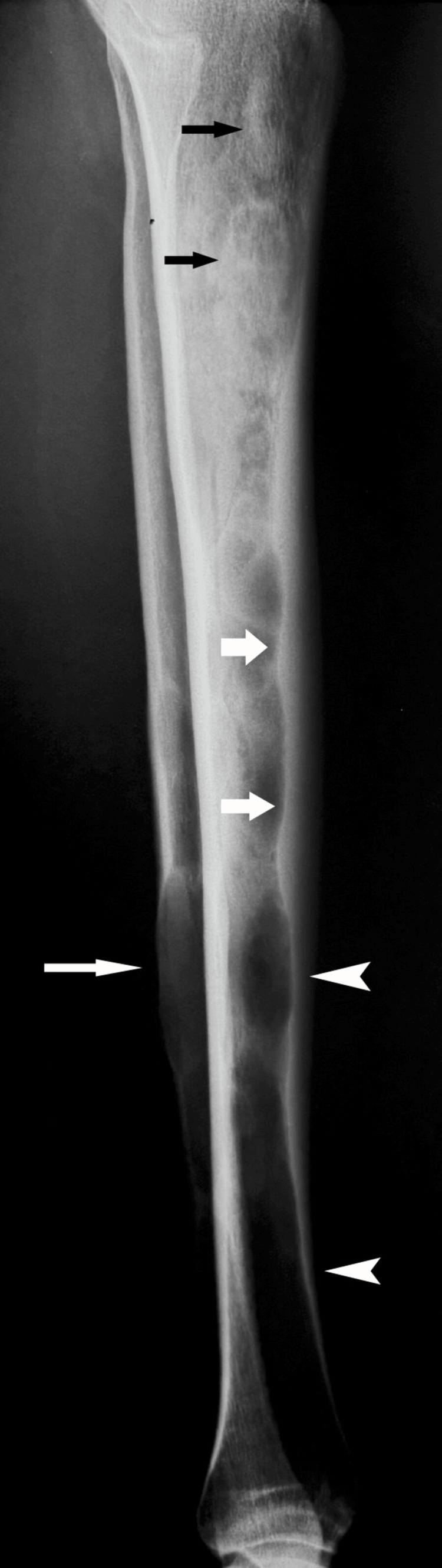
Lateral radiograph of the lower limb demonstrates multiple diaphyseal radiodense (arrows) and radiolucent lesions (arrowheads) in the tibia and fibula. Cortical scalloping (thick arrows) is seen. Note the large, lytic expansile lesion (long arrow) within the diaphysis of the distal fibula.

**Figure 2:**
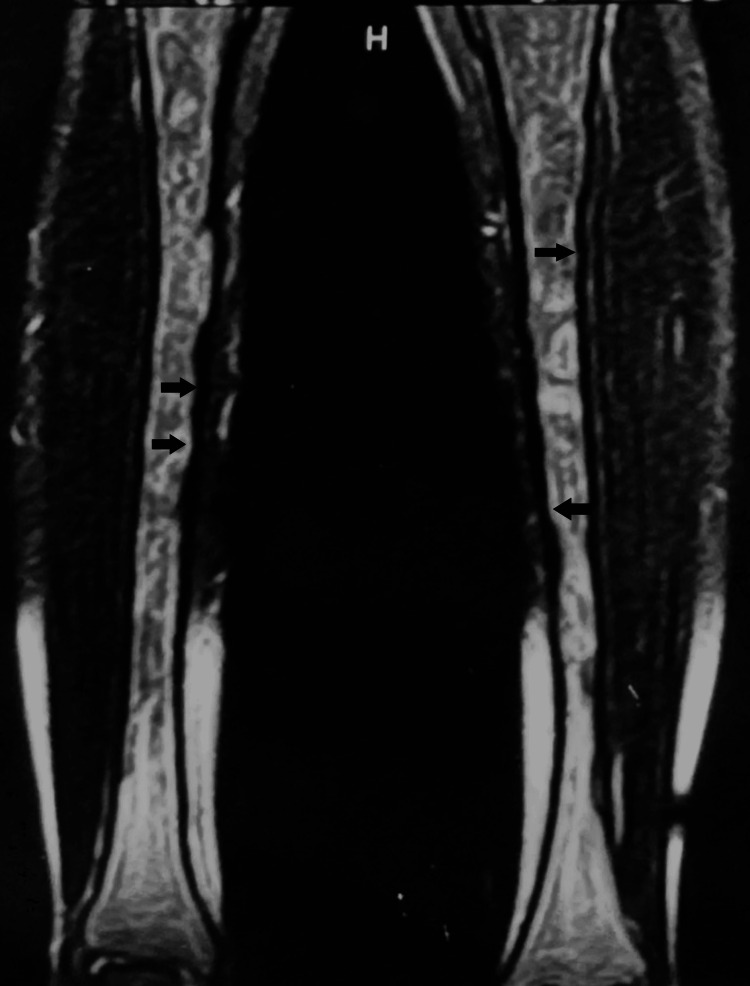
Coronal T2-weighted MR image shows diffuse, markedly heterogeneous signal intensity in tibial diaphysis on both sides. Note endosteal scalloping (arrows) without cortical disruption.

**Figure 3:**
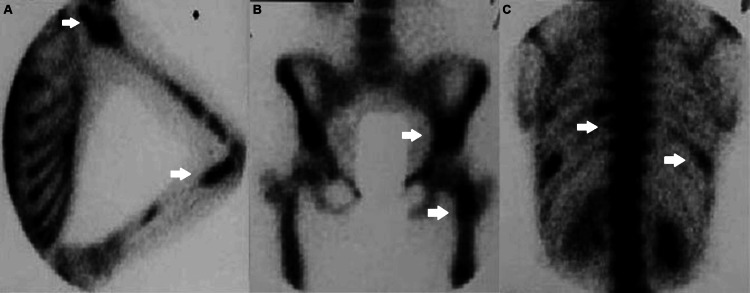
Bone scan (A-C) shows multiple foci of increased radionuclide uptake throughout the examined skeleton. Foci of increased radionuclide uptake at (A) left arm and forearm, (B) left hip and pelvis, and (C) thoracolumbar section of the spine and 10th rib

**Figure 4:**
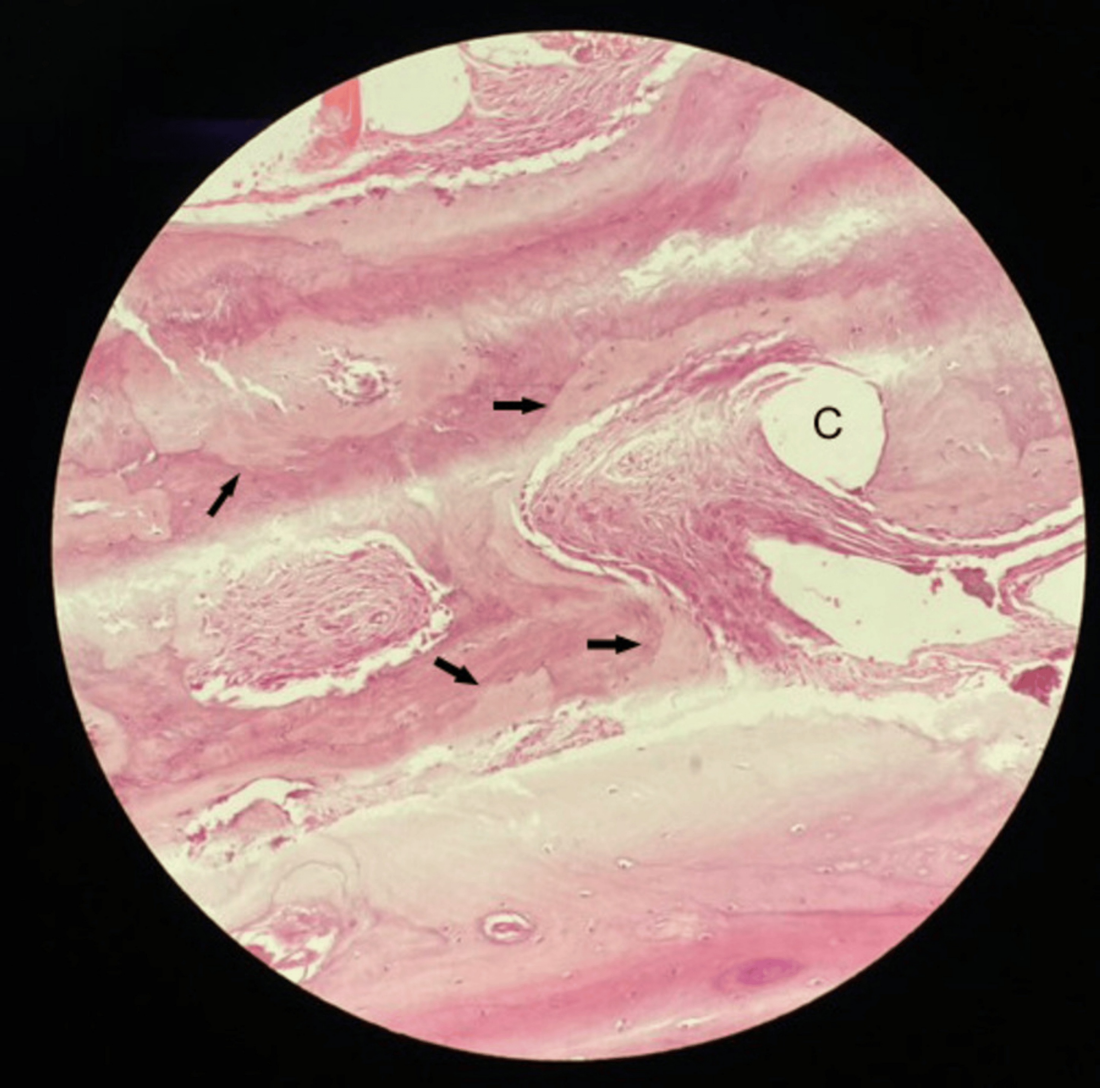
Histological specimen of lesion in the fibula shows varying degrees of osseous and fibrous tissue components, with irregular trabeculae (arrows) in a fibrous background. Some cystic changes (C) are present.

The female patient, now 46 years old, had undergone radiological examinations at age 23 for intense pain in the left leg without a history of trauma. Radiographs demonstrated areas of radiolucency with a characteristic "ground glass" appearance, indicative of fibrous dysplasia (Figure 1). MR images displayed lesions of heterogeneous signal intensity in the diaphysis of both tibiae alternating with areas of low and high signal intensity and uneven thickening of (non-disrupted) cortical bone, consistent with fibrous dysplasia (Figure 2). Blood and urine tests were normal. Bone scans showed multiple lesions with radionuclide uptake in the long bones of the upper and lower extremities, the trunk, and the skull (Figure 3). A bone biopsy of a lesion in the fibula confirmed the diagnosis of fibrous dysplasia (Figure 4).

**Figure 5:**
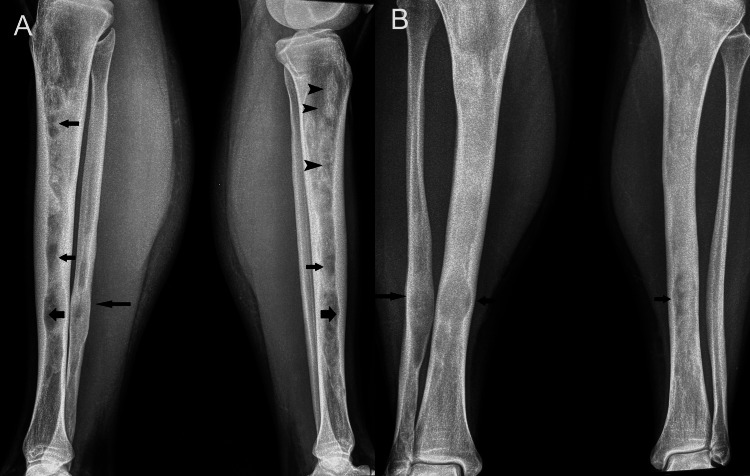
Oblique and lateral (A) and anteroposterior (B) radiographs of the tibia and fibula on both sides show extensive alterations of polyostotic fibrous dysplasia featuring multiloculated, mixed radiolucent (arrows) and radiodense (arrowheads) lesions involving the entire shaft of the bones. Associated endosteal scalloping (thick arrows) and cortical thinning are seen. Note focal bone expansion mostly in the fibula (long arrow).

The patient was reevaluated once a year for the following three years. On radiographs (Figure 5) and blood tests, there was no appreciable differentiation as compared to the examinations at the initial presentation.

**Figure 6:**
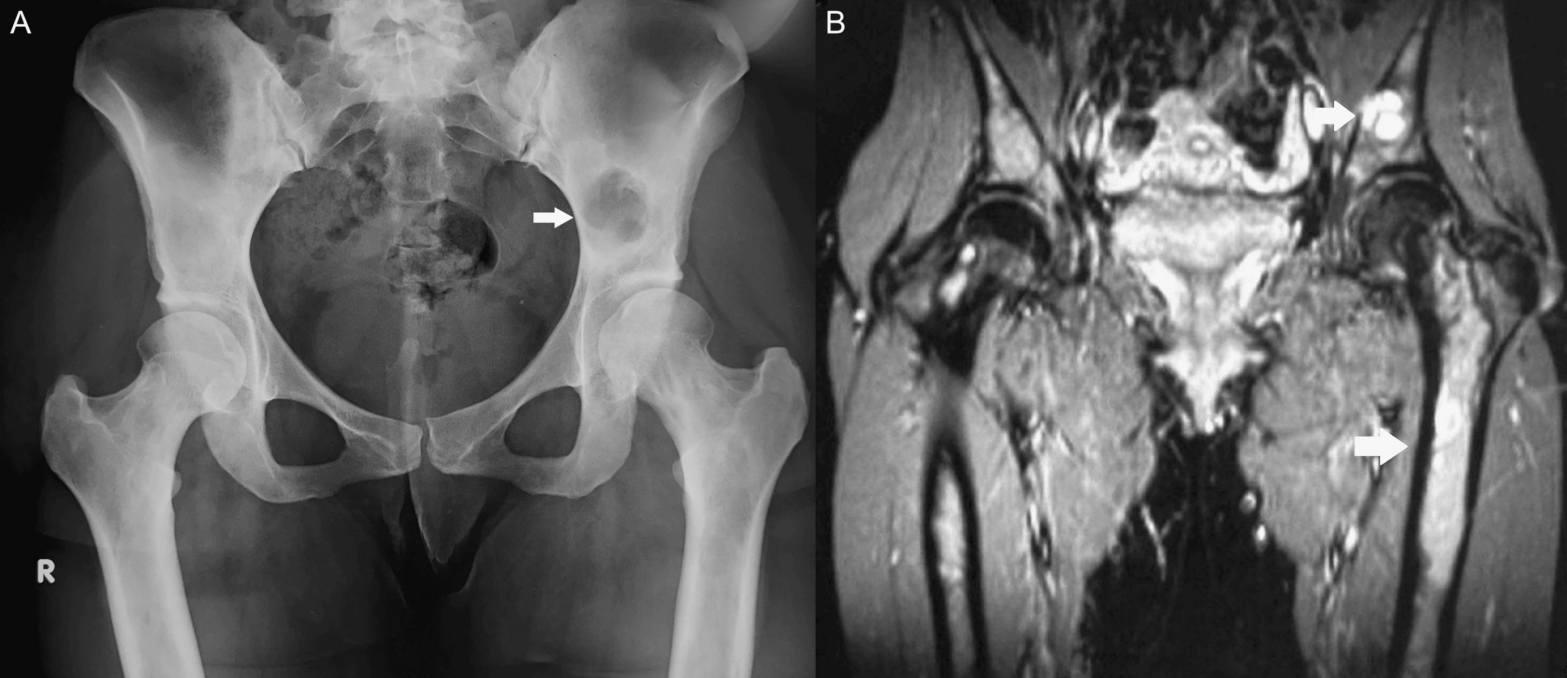
Frontal radiograph (A) and coronal STIR MR image (B) of the pelvis and hips. Shows a prominent FD lesion (arrow) in the left iliac bone, and (B) reveals extensive FD lesions involving the femora and the iliac bones. There is also moderate expansion of bone in the left femur. STIR MRI: Short Tau Inversion Recovery MRI

Four years after the initial evaluation, the patient reported the onset of severe left hip pain and discomfort during everyday tasks. Radiographs and MR images of the pelvis and hips (Figure 6) showed enlargement of FD lesions in the left ilium and the femora. Laboratory examinations showed hypophosphatemia (2.39 mg/dl, with normal range 2.5-5.5 mg/dl), increased serum level of osteocalcin (60.50 mg/ml, with normal range 4-32 ng/ml), and high levels of hydroxyproline (29.70 mg/24h, with normal range 6-22 mg/24h) in the 24-hour urine sample.

**Figure 7:**
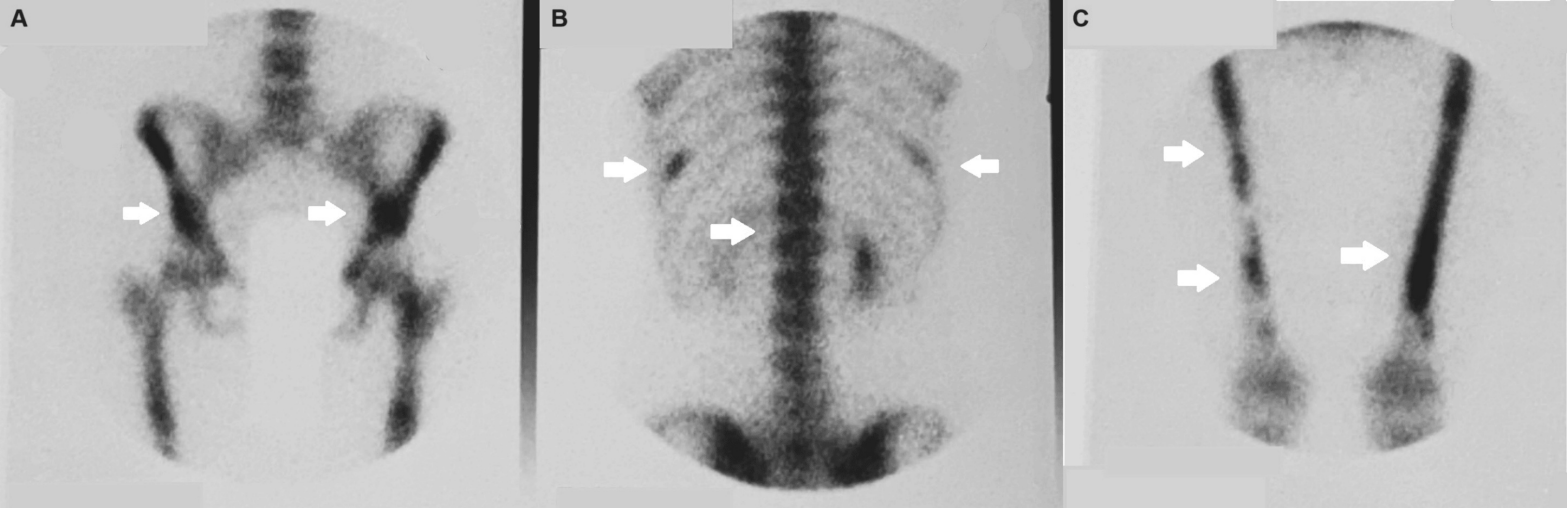
The second bone scan (A-C) shows again multiple foci of increased radionuclide uptake throughout the examined skeleton. Foci of increased radionuclide uptake at (A) right and left iliac bones, (B) thoracolumbar section of the spine and on the ribs, and (C) on the diaphyses of both femurs

A repeated bone scan (Figure 7) revealed multiple foci of increased uptake in the pelvis, the 10th rib on both sides, the long bones in the lower and upper extremities, and the skull, along with diffusely increased uptake in the vertebrae and the sternum. These findings were compatible with the development of multiple new skeletal FD lesions.

Over the next few years, the patient was treated conservatively with analgesics and regular physiotherapy sessions. On radiographs and MR imaging examinations, new FD lesions were seen at the distal humerus and the proximal radius.

**Figure 8:**
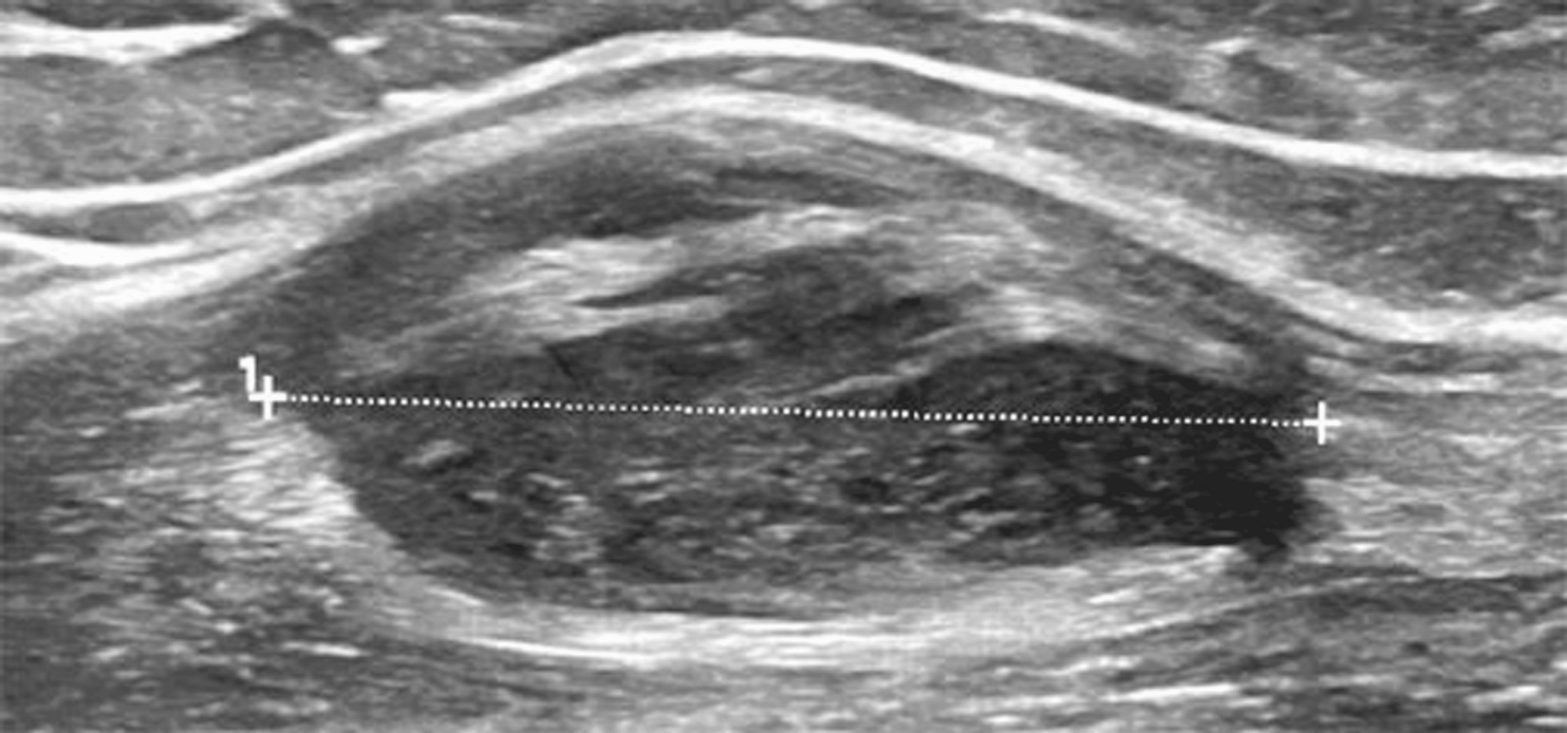
Ultrasound of palpable mass in the left arm shows spindle-shaped intramuscular lesion measuring 27 mm.

Fifteen years after the initial diagnosis of FD, at the age of 38, the patient presented at the orthopedic outpatient clinic with a palpable mass located on the anterior surface of her left arm. The patient reported that she had been experiencing discomfort for several weeks but initially disregarded it as muscle strain from her routine activities. Due to persistent pain associated with the soft tissue mass, she underwent a new imaging evaluation. Radiographs of the upper and lower limbs at this time displayed the imaging findings of polyostotic fibrous dysplasia, with no apparent interval changes. Ultrasound examination of the left arm demonstrated an oval-shaped soft tissue mass, with a maximum length of 9 cm and a width of 2.7 cm. The mass was hypoechoic, well-defined, with no significant internal vascularity, raising suspicion for a benign soft tissue tumor (Figure 8).

**Figure 9:**
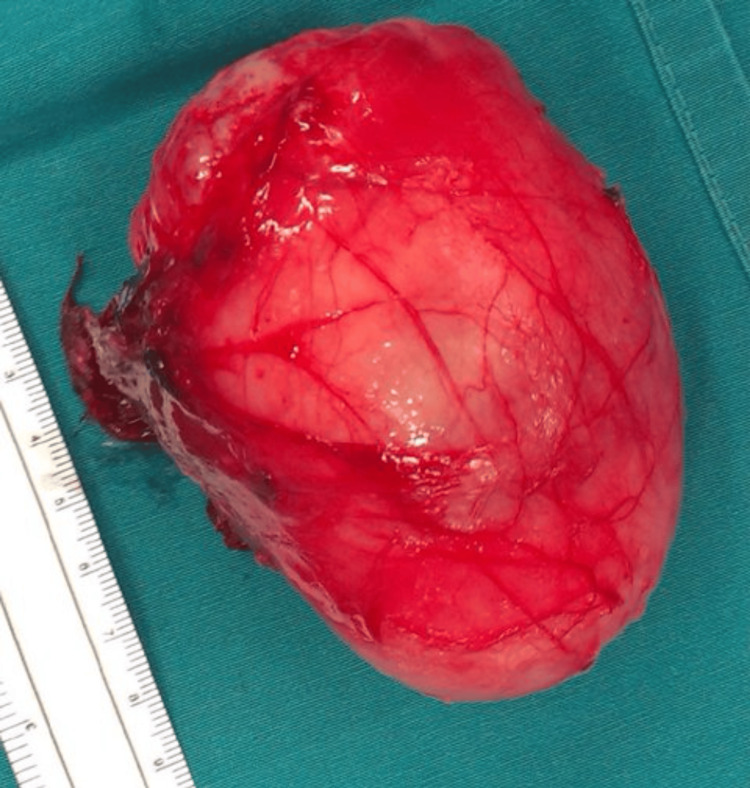
The excised myxoma of the left arm.

Surgical excision of the mass was performed (Figure 9), and the specimen was sent for histological analysis. Histopathological examination revealed a myxomatous lesion consistent with intramuscular myxoma. This finding, in conjunction with the imaging characteristics of FD, led to the diagnosis of Mazabraud syndrome, 15 years after the initial examination of the patient. Unfortunately, because the patient's health insurance could not cover the expenses, guanine nucleotide-binding protein, alpha stimulating (GNAS) genetic testing was not performed. Instead, it had to be done at private laboratories, which the patient declined since she lacked the necessary financial resources.

For the following three years after the excision of intramuscular myxoma, the patient was subject to follow-up, with no new findings.

**Figure 10:**
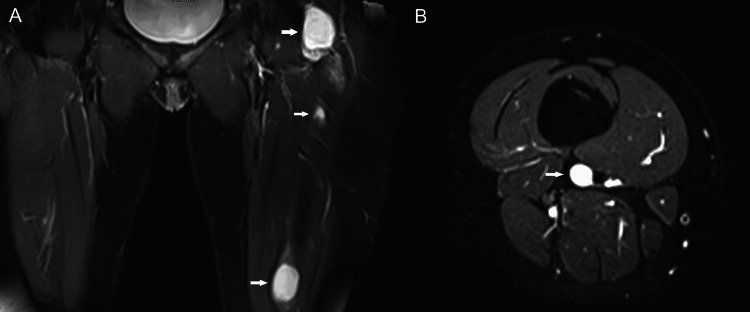
Coronal (A) and axial (B) STIR MR images depict myxomas (arrows) of high signal intensity in musculature of the left thigh.

**Figure 11:**
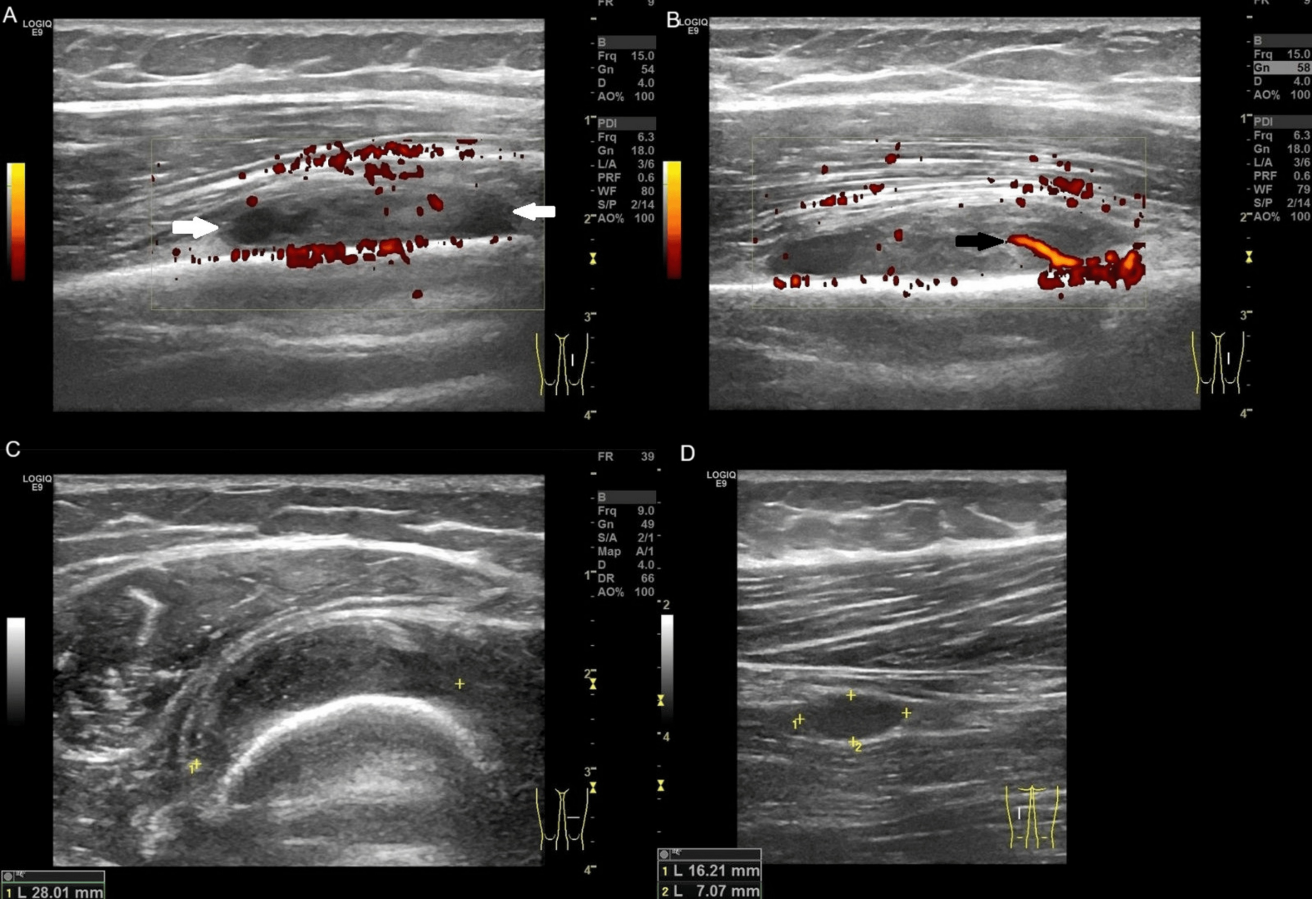
Ultrasound images of the left thigh. Longitudinal color Doppler (A-B) ultrasound images of the thigh show a fusiform, heterogeneous soft tissue mass with peripheral vascularity. A feeding vessel is seen (B, black arrow). Note that echogenicity is markedly lower at the poles of the mass (A, white arrows). Transverse ultrasound images (C-D) show an intramuscular mass of mixed, mostly low echogenicity, in the thigh.

Nevertheless, during the long follow-up time (18 years after the initial diagnosis of FD and three years after the diagnosis of myxoma), the patient at age 41 presented with a new, palpable mass on her left thigh. The mass was painless, and it was growing in the quadriceps muscle over several months. In addition to the known bone FD lesions, MR imaging displayed three well-defined intramuscular lesions of high signal intensity on the T2 and STIR sequences in the thigh (Figure 10). On contrast-enhanced MR images, the lesions demonstrated mild contrast enhancement. Ultrasound depicted the well-defined hypoechoic to near-anechoic mass lesions with some internal echoes (Figure 11). In one of these lesions, a rim of increased echogenicity was seen around the mass, corresponding to adipose tissue at the periphery of the lesion. Color Doppler ultrasound showed the lesions to be hypovascular. 

Because the imaging findings of the oval-shaped soft tissue mass lesions located within the thigh musculature were those of intramuscular myxomas, a biopsy was not performed. In our patient, the presence of multiple myxomas in association with polyostotic FD was diagnostic for Mazabraud syndrome. Given the benign nature of myxomas and the absence of functional or aesthetic concerns, conservative treatment was pursued with the patient's consent. The patient was informed of the likelihood of malignant degeneration of the lesions and the importance of regular follow-up to monitor changes over time.

## Discussion

Mazabraud syndrome is a rare clinical entity characterized by the concurrence of FD with intramuscular myxomas [1,2]. The pathophysiology underlying this syndrome remains unclear, but it raises intriguing questions about the genetic and environmental factors that may contribute to the development of both conditions. The literature suggests a potential association between FD and the development of myxomas, although the exact mechanisms are not fully understood [10].

Fibrous dysplasia is associated with mutations in the GNAS gene, which encodes the alpha subunit of the stimulatory G protein [11,12]. This mutation relates to abnormal osteoblastic activity with the replacement of normal bone by fibrous tissue, resulting in the characteristic radiographic "ground-glass" appearance of affected bones. The clinical manifestations of FD can vary widely, with some patients remaining asymptomatic while others experience pain, deformities, and an increased risk of pathological fractures [13]. The polyostotic form of FD, however, is often associated with syndromic conditions, such as McCune-Albright syndrome, comprising precocious puberty, café-au-lait skin spots, and endocrine abnormalities [14]. Gun et al. suggested that FD is usually associated with hypophosphatemia, which was also present in our case [15].

In patients with FD, the coexistence of intramuscular myxoma, a benign soft tissue tumor characterized by the proliferation of myxoid connective tissue, has been adequately documented [16]. Myxomas present as painless, slow-growing mass lesions, usually in the thighs and less frequently in the buttocks and upper arms, with a 57% female predilection [4,17-19]. As with all myxoid soft tissue lesions, myxomas may need to be differentiated from other benign and malignant soft tissue tumors, including lipomas, ganglion cysts, or sarcomas. Few articles in the literature have reported on the malignant transformation (osteosarcoma, fibrosarcoma) of FD lesions in Mazabraud syndrome [20,21]. Because of the benign behavior in most cases, management options include regular observation or surgical excision with a curative intent. After the initial excision of a solitary myxoma in the arm, we elected to closely observe recurrent myxomas in the lower limbs of our patient with polyostotic FD.

Because Mazabraud syndrome is rare, diagnosis can be challenging [22]. In this regard, imaging plays a key role in the evaluation of the condition that may help differentiate benign and malignant lesions. Indeed, MR imaging is considered the imaging study of choice that allows for accurate diagnosis of patients with Mazabraud syndrome [23], featuring the combined imaging findings of two distinct abnormalities: FD and myxoma. FD lesions demonstrate a decreased signal intensity on T1-weighted images and increased signal intensity on T2-weighted MR images. A border of decreased signal intensity is noted at the periphery of the lesions at all sequences [4]. On contrast-enhanced MR images, FD lesions usually demonstrate moderate heterogeneous contrast enhancement. Myxomas demonstrate homogeneous low to intermediate signal intensity on T1-weighted MR images and high signal intensity on T2-weighted MR images [16]. In our patient, the imaging appearances of multiple intramedullary bone lesions with well-defined borders, combined with oval-shaped intramuscular soft tissue mass lesions, were most consistent with Mazabraud syndrome [16,23,24]. Surgical excision of the myxoma and conservative treatment of FD were considered appropriate because of the benign behavior of lesions.

Despite a known benign biologic behavior, however, clinicians managing patients with FD need to be acquainted with the imaging presentations of Mazabraud syndrome, as early and correct diagnosis can guide appropriate management [25]. Although conservative management of FD is typical, intramuscular myxomas may warrant surgical intervention, particularly if lesions cause functional impairment or cosmetic concerns. For example, in a previous study, Li et al. reported two cases of Mazabraud syndrome where differentiation of myxomatous lesions from malignant tumors was important to avoid aggressive excision of the myxoid soft tissue lesions [26].

Although sporadic cases of Mazabraud syndrome have been reported in medical literature, the pathogenesis and natural history of the disease and the biological potential for complications have not been fully elucidated. Further research is needed to explain a possible link between bone lesions and soft tissue myxomas. Previous investigators have found that the identification of intramuscular myxomas may indicate a more widespread form of FD [27]. These findings and the implications for management warrant further investigation. Our case contributes to the growing body of literature on Mazabraud syndrome, reporting on the long-term follow-up (23 years for FD and eight years for myxomas) of a patient with FD who eventually (15 years after initial diagnosis of FD) developed intramuscular myxomas.

## Conclusions

Mazabraud syndrome is a unique clinical entity characterized by the rare coexistence of FD and intramuscular myxoma. This case underscores the clinical significance of recognizing Mazabraud syndrome in patients with FD, as early identification can facilitate appropriate management strategies. The benign nature of both FD and intramuscular myxomas is associated with a favorable prognosis; however, continued surveillance is warranted to monitor potential complications, such as malignant transformation or the development of additional myxomas. Further research is needed to better understand the underlying mechanisms and long-term outcomes associated with Mazabraud syndrome, as well as to improve diagnostic accuracy and treatment protocols for affected patients. Clinicians should maintain a high index of suspicion for Mazabraud syndrome in patients with FD and soft tissue masses, as timely intervention can prevent unnecessary morbidity and improve overall patient outcomes.
